# Anoctamin 1 contributes to inflammatory and nerve-injury induced hypersensitivity

**DOI:** 10.1186/1744-8069-10-5

**Published:** 2014-01-23

**Authors:** Byeongjun Lee, Hawon Cho, Jooyoung Jung, Young Duk Yang, Dong-Jin Yang, Uhtaek Oh

**Affiliations:** 1 Sensory Research Center, CRI, College of Pharmacy, Seoul National University, Gwanak, Daehak-Ro 1, Seoul 151-742, Republic of Korea; 2 Department of pharmacy, College of Pharmacy, CHA University, Gyeonggi-do, Republic of Korea

**Keywords:** ANO1, Neuropathic pain, Inflammatory pain, DRG neuron, Hyperalgesia, Rheobase

## Abstract

**Background:**

Various pathological conditions such as inflammation or injury can evoke pain hypersensitivity. That represents the response to innocuous stimuli or exaggerated response to noxious stimuli. The molecular mechanism based on the pain hypersensitivity is associated with changes in many of ion channels in dorsal-root ganglion (DRG) neurons. Anoctamin 1 (ANO1/TMEM16A), a Ca^2+^ activated chloride channel is highly visible in small DRG neurons and responds to heat. Mice with an abolished function of ANO1 in DRG neurons demonstrated attenuated pain-like behaviors when exposed to noxious heat, suggesting a role in acute thermal nociception. In this study, we further examined the function of ANO1 in mediating inflammation- or injury-induced hyperalgesia or allodynia.

**Results:**

Using *Advillin/Ano1*^*fl/fl*^ (*Adv/Ano1*^*fl/fl*^) mice that have a functional ablation of *Ano1* mainly in DRG neurons, we were able to determine its role in mediating thermal hyperalgesia and mechanical allodynia induced by inflammation or nerve injury. The thermal hyperalgesia and mechanical allodynia induced by carrageenan injection and spared-nerve injury were significantly reduced in *Adv/Ano1*^*fl/fl*^ mice. In addition, flinching or licking behavior after bradykinin or formalin injection was also significantly reduced in *Adv/Ano1*^*fl/fl*^ mice. Since pathological conditions augment nociceptive behaviors, we expected ANO1′s contribution to the excitability of DRG neurons. Indeed, the application of inflammatory mediators reduced the threshold for action potential (rheobase) or time for induction of the first action potential in DRG neurons isolated from control (*Ano1*^*fl/fl*^) mice. These parameters for neuronal excitability induced by inflammatory mediators were not changed in *Adv/Ano1*^*fl/fl*^ mice, suggesting an active contribution of ANO1 in augmenting the neuronal excitability.

**Conclusions:**

In addition to ANO1's role in mediating acute thermal pain as a heat sensor, ANO1 is also capable of augmenting the excitability of DRG neurons under inflammatory or neuropathic conditions and thereby aggravates inflammation- or tissue injury-induced pathological pain.

## Introduction

Pain is one of many diverse sensations that humans experience in life. Pain is an unpleasant sensation that evokes suffering in humans. However, pain is also a warning sign for avoiding tissue injury from impending external noxious stimuli or aggravated pathological conditions. Acute pain that is mediated by noxious stimuli from external environments functions as a warning system that notifies the risk of immediate injury [1,2]. In contrast, inflammation, nerve damage, or cancer causes pain hypersensitivity or exaggerated responses to non-noxious stimuli injury [1,2]. Pain is initiated with the detection of noxious stimuli at terminals of DRG neurons innervated in peripheral nervous system. Nociceptor is a subclass of DRG neurons that is especially for transmitting nociceptive neural signals. Most of nociceptors are small-diameter and unmyelinated c-fibers [3]. Nociceptors express many ion channels that are associated with pain signal generation. Many transient receptor potential (TRP) channels present in DRG neurons are involved in transducing noxious stimuli to neural signals [4,5]. In addition to their role as a primary detector for noxious stimuli, these channels also play a role in transmitting nociceptive signals to the spinal cord, amplifying nociceptive neural signals. A good example is TRPV1, which is activated by diverse stimuli such as capsaicin, anandamide, lipoxygenase products, noxious heat or low pH [6-9]. TRPV1 is highly visible in nociceptors of DRG neurons and known to play an important role in transducing these noxious stimuli to nociceptive neural signals. Because *Trpv1*^-/-^ mice failed to detect capsaicin-evoked pain and showed reduced response to heat, TRPV1 is now known to be a heat sensor that detects noxious heat [9]. In addition, inflammation- and nerve injury-induced thermal hyperalgesia were also reduced in *Trpv1*^-/-^ mice, suggesting a role for controlling transmission of nociceptive signals [9,10]. Similarly, TRPA1 is activated by pungent chemicals or environmental irritants [11,12]. TRPA1 is expressed in nociceptive neurons that are positive to TRPV1 or CGRP. In addition, bradykinin (BK), an algogenic substance released by tissue damage, stimulates TRPA1 [11]. TRPA1 appears to contribute to cold hyperalgesia or allodynia after inflammation and nerve injury, because TRPA1 knock down evoked the reduction of cold hyperalgesia [13-15]. Thus, many channels in DRG neurons play a role in detecting noxious stimuli as primary sensors and are actively involved in modulating transmission of the nociceptive signals to the central nervous system.

ANO1 is a Cl^-^ channel activated by intracellular Ca^2+^[16-18]. ANO1 as a Ca^2+^-activated Cl^-^ channel mediates transepithelial fluid secretion in the epithelia of many organs [19]. Notably, ANO1 is implicated in salivation [20], mucin secretion in airway [19], intestinal function [21], and vascular tone [22]. In addition, Yang et al. reported that ANO1 is expressed in DRG neurons, suggesting a role in somatosensation [16]. A later study on the expression in DRG neurons revealed that ANO1 is highly co-localized with TRPV1, suggesting a role in nociception [23]. Surprisingly, ANO1 is activated by heat over 44°C [23]. Functional ablation of ANO1 in DRG neurons elicited significant loss of thermal pain [23]. Although these results suggest that ANO1 plays a role in transducing noxious heat as a heat sensor, it is not known whether ANO1 is implicated in controlling excitability of sensory neurons, which mediates hyperalgesia or allodynia evoked by chronic tissue injury. Thus, we sought to determine ANO1′s roles in regulating hyperalgesia or allodynia evoked by pathological conditions such as inflammation or nerve injury.

## Methods

### Animals

This study was performed in accordance with protocols approved by the Committee on Laboratory Animals at Seoul National University. Experiments were also conducted according to the Ethical Guidelines of the International Association for the Study of Pain. We crossed *Adv*^cre/WT^*Ano1*^fl/fl^ homozygote with *Adv*^WT/WT^*Ano1*^fl/fl^ homozygote, producing *Adv*^cre/WT^*Ano1*^fl/fl^ and *Adv*^WT/WT^*Ano1*^fl/fl^ offsprings. *Adv*^cre/WT^*Ano1*^fl/fl^ mice were used as conditional knock-out mice (*Adv/Ano1*^fl/fl^) whereas *Adv*^WT/WT^*Ano1*^fl/fl^ homozygote were used as a ‘control’ mice (*Ano1*^fl/fl^). Adult (18 – 23 g) *Adv/Ano1*^fl/fl^_,_ and *Ano1*^fl/fl^ mice were used for the behavioral tests. All experiments were performed during the day time between the hours (9:00 a.m. and 6:00 p.m.). Animals for all experiments were habituated before testing.

### Spared-nerve injury (SNI)-induced neuropathic pain

Neuropathic pain was induced in mice with nerve injury as described [24]. Briefly, mice were anesthetized by intraperitoneal (i.p.) injection of 50 mg/kg pentobarbital. We checked reflexes by pinching paws using a pincette to test whether mice were under anesthetic condition. An incision (~0.3 cm long) around the knee was made in the longitudinal direction. Nerve injury was induced through cutting tibial and common peroneal-nerve branches among three branches of the sciatic nerve.

### Hargreaves plantar test

This test was performed using a standard apparatus (Ugo Basile Biological research Apparatus) as previously described [25]. Briefly, a mouse was placed in a transparent acrylic box. An infrared heat lamp was positioned underneath the targeted hind paw. A radiant stimulus (intensity 50) was then applied to the plantar surface. Each test was repeated three times with 5 min interval. The paw withdrawal latency was calculated as the average of three values measured.

### Von Frey hair test

Mechanical allodynia was measured by prodding the plantar region of the hindpaw with calibrated von Frey filaments (Stoelting Co., Wood Dale, IL). Animals were placed in cages with a mesh grid floor. On testing day, mice were allowed to acclimate for a minimum of 30 min before the experiment. Plantar surface of the hind foot was poked with Von Frey filaments of different thickness. Withdrawal thresholds to Von Frey filaments were determined when animals lifted their hindpaw at least 5 responses out of 10 stimulations. Minimum weight of the Von Frey filament that evoked a response was considered as a mechanical withdrawal threshold.

### Bradykinin and formalin induced nocifensive behavior test

Mice were placed in a transparent acrylic box and allowed to acclimate for approximately 1 hr before injection. Nocifensive behaviors of mice were measured for 30 min after bradykinin injection. Formalin-induced behaviors were evaluated for 45 min being subdivided into two phases (phase I; 0-15 min, phase II; 15–45 min). Behaviors of mice such as biting, flinching or licking were considered as nocifensive behaviors.

### Drug treatment

To induce inflammation by carrageenan, carrageenan (2%) was injected subcutaneously into the plantar surface of left hind paws of mice (50 μl). Bradykinin (1 μg) and formalin (5%, 50 μl) were administered subcutaneously into plantar surfaces of left hind paws of control and *Adv/Ano1*^*fl/fl*^ mice. The inflammatory soup contained 10 μM of bradykinin, prostaglandin E_2_, serotonin, histamine, respectively. The inflammatory soup was applied to DRG neurons 2 hr before current clamp recordings. All chemicals were purchased from Sigma-Aldrich and dissolved in physiological saline.

### Culture of primary DRG neuron

Primary cell cultures of DRG neurons were conducted as previously described [23]. Thoracic and lumbar DRGs were dissected from control and *Ano1/Adv*^*fl/fl*^ mice, and collected in cold culture medium (4°C) containing a mixture of DMEM and F-12 solution, 10% fetal bovine serum (Gibco BRL), 1 mM sodium pyruvate, 50–100 ng/ml nerve growth factor (Alomon, Jerusalem, Israel), and 100 units/ml of penicillin/streptomycin. Ganglia was washed with culture medium and incubated for 30 min in a warm (37°C) DMEM/F-12 mixture containing 1 mg/ml of collagenase (Type II; Worthington Biomedical). DRGs were then washed 3 times with Mg^2+^- and Ca^2+^-free Hank’s solution, and incubated while gently shaking in Hank’s solution containing 2.5 mg/ml of trypsin (Roche Diagnostics) for 30 min at 37°C. The trypsin-containing solution was then centrifuged at 100 g for 10 min. The pellets obtained after centrifugation were washed gently 2–3 times with culture medium and gently triturated with a fire-polished Pasteur pipette. Cells were plated onto round glass coverslips (Fisher), which had been previously treated with poly-L-lysine (0.5 mg/ml), in small Petri dishes (35 × 12 mm). Cells were then placed in a 37°C incubator in a 95% air/5% CO_2_ atmosphere. Cells were used 2–4 days after plating.

### Electrophysiology

Current clamp recordings were performed using an Axopatch 200B (Molecular devices). Data was amplified, stored in a personal computer after digitization using Digidata 1440 (Molecular devices). Bath solution contained 140 mM NaCl, 5 mM KCl, 10 mM HEPES, 2 mM MgCl_2_, 2 mM CaCl_2_, adjusted to pH 7.2. Pipette solution contained 30 mM KCl, 100 mM K-aspartate, 10 mM HEPES, 1 mM MgCl_2_, 1 mM EGTA, and 0.225 mM CaCl_2_ adjusted to pH7.2. Whole cells were formed after breaking the plasma membrane under pipette tips. Resistance of the glass pipette was about 3 MΩ. Junctional potentials were cancelled to zero. The recordings were switched to the current clamp configuration. After a stable baseline was recorded, resting membrane potential (RMP) was measured in the DRG neurons of inflammatory soup treated group and vehicle treated group. The rheobase was recorded by an injection of current from 50 pA to 1 nA with 50 pA interval. Current injection with 1 nA magnitude was used to measure the latency to evoke first action potential spike.

### Statistics

All results are expressed as means ± SEMs and were analyzed by Student-*T* test. One way ANOVA was used for multiple comparison of the means followed by Duncan’s post-hoc test. Statistical significance was accepted for p values of < 0.05.

## Results

### Reduction in inflammatory hyperalgesia and allodynia in Adv/Ano1^fl/fl^*mice*

**Figure 1:**
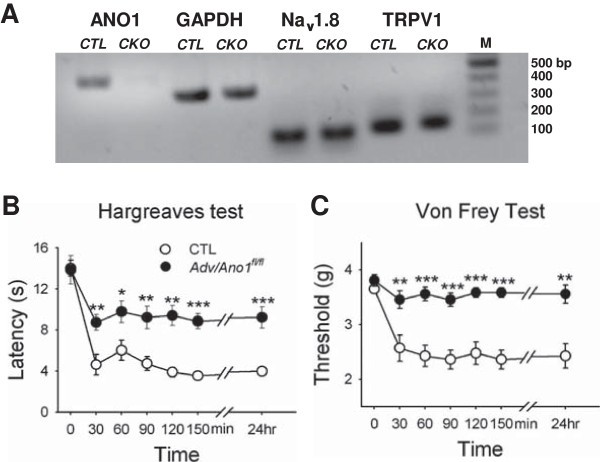
**Tissue specific ablation of *Ano1 *in DRG neurons reduces inflammatory thermal and mechanical hyperalgesia. (A)** Detection of ANO1, Nav1.8 and TRPV1 mRNAs in mouse DRG neurons of *Ano1*^*fl/fl*^ (control; CTL) and *Adv/Ano1*^*fl/fl*^ (conditional knockout; CKO) mice using reverse transcription-PCR. M, DNA molecular weight markers. **(B)** Time courses of the withdrawal latency to thermal stimuli were examined in *Adv/Ano1*^*fl/fl*^ and its control mice 30, 60, 90 120, 150 min, 24 hr after carrageenan injection (2%, 50 μl). Radiant heat stimulus (IR 50) was applied to left paws of *Adv/Ano1*^*fl/fl*^ and its control mice (n = 7–8, *p < 0.05, **p < 0.01, ***p < 0.001). Basal withdrawal latency was measured before the injection of carrageenan. **(C)** Time courses of withdrawal thresholds of *Adv/Ano1*^*fl/fl*^ and its control mice to Von Frey hair stimuli 30, 60, 90 120, 150 min, 24 hr after carrageenan injection. Mechanical stimuli were applied to left hind paws of *Adv/Ano1*^*fl/fl*^ and its control mice with Von Frey hairs (n = 6, **p < 0.01, ***p < 0.001). Basal withdrawal threshold was measured before the injection of carrageenan.

We first investigated whether tissue specific knocked out of *Ano1* in DRG neurons has an effect on inflammatory hyperalgesia or allodynia. To disrupt functional ANO1 in DRG neurons, *Ano1*^fl/fl^ mice were crossed with *advillin-cre* transgenic mice (see Methods). Advillin is a member of the gelsolin family, actin binding proteins and known to be present exclusively in DRG neurons [26]. The generation of *Adv/Ano1*^*fl/fl*^ mice was confirmed as described previously [23]. In DRG neurons from *Adv/Ano1*^*fl/fl*^ mice, *Ano1* transcripts were absent whereas Nav1.8 and TRPV1 transcripts were not changed (Figure 1A). To induce inflammation in a hind paw, carrageenan (2% w/v, 50 μl; s.c.) was injected into the plantar surface of a left hind paw of a mouse. The withdrawal latency from radiant heat was measured at time points of 30, 60, 90, 120, 150 min, and 24 hours after carrageenan administration (Hargreaves test). As shown in Figure 1B, apparent significant decrease (p < 0.001, one way-ANOVA, Duncan post-hoc test) in the withdrawal latency was observed in control mice from 30 min after carrageenan injection. The reduction in withdrawal latency after administration of carrageenan was sustained at all time-points tested up to 24 hr. A significant decrease (p < 0.05, one way-ANOVA, Duncan post-hoc test) in the withdrawal latency for radiant heat was also observed in *Adv/Ano1*^*fl/fl*^ mice. However, the reduction in withdrawal latency in *Adv/Ano1*^*fl/fl*^ mice was significantly less than that of control mice at all time points (p < 0.001 ~ 0.05, n = 7–8) (Figure 1B).

To see if the ablation of *Ano1* in DRG neurons also affects mechanical allodynia, the Von Frey-hair test was conducted after the injection of carrageenan to hind paws of *Adv/Ano1*^*fl/fl*^ mice. A significant drop (p < 0.01, one way-ANOVA, Duncan post-hoc test) in the withdrawal threshold for mechanical allodynia was observed in control mice from 30 min after carrageenan injection (Figure 1C). In contrast, this drop in the withdrawal threshold for mechanical allodynia after carrageenan injection was not observed in *Adv/Ano1*^*fl/fl*^ mice (p < 0.01 ~ 0.001, n = 6). Thus, *Adv/Ano1*^*fl/fl*^ mice exhibited significantly less thermal hyperalgesia and mechanical allodynia induced by inflammation.

### ANO1 reduces nociception evoked by bradykinin and formalin

**Figure 2:**
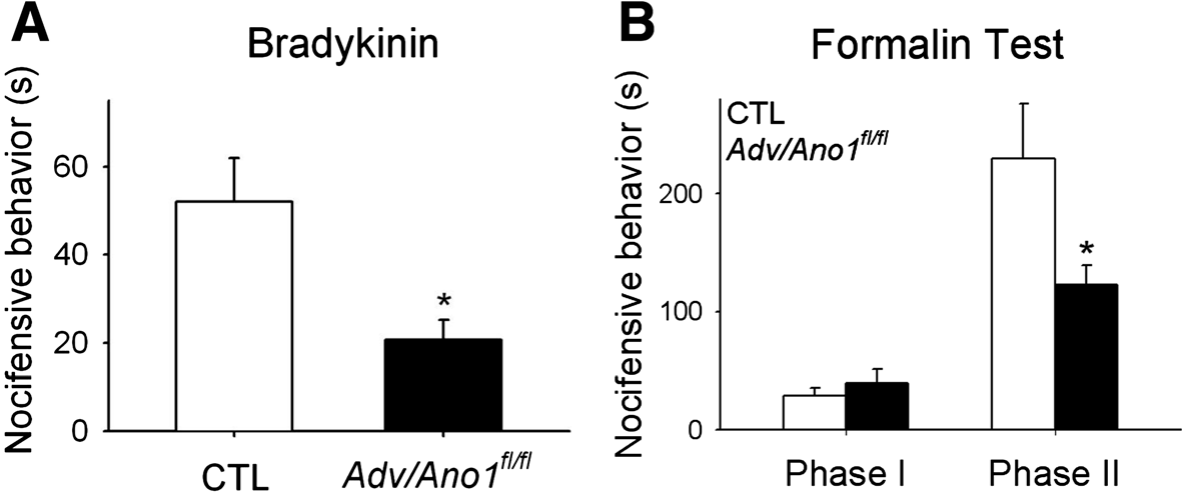
**Ablation of ANO1 in DRG neurons attenuates bradykinin and formalin induced nociception. (A)** Nocifensive behaviors were counted for 30 min after bradykinin (1 μg, 50 μl) was injected to left hind paws of *Adv/Ano1*^*fl/fl*^ and its control mice (*p < 0.05, n = 8). **(B)** Nocifensive behaviors were counted for 45 min after formalin (5%, 50 μl) was injected to left hind paws of *Adv/Ano1*^*fl/fl*^ and its control mice (*p < 0.05, n = 6–7). Phase I represents measurement for the first 15 min while phase II represents measurement for 15 ~ 45 min.

BK has been known as a potent algogenic substance that is released when tissue is injured [27,28]. In addition, BK is known to induce nocifensive behaviors via activation of CaCCs because BK-induced nocifensive behaviors were reduced by CaCC blockers in rats [29]. Therefore, we attempted to confirm whether ANO1 mediates BK-evoked nociception. The subcutaneous injection of 1 μg BK into the plantar surface of hind paws of the control mice induced nocifensive behaviors for 52 ± 11 seconds (n = 8) (Figure 2A). However, these nocifensive behaviors after BK injection lasted only 20.7 ± 4.9 seconds (n = 8) in *Adv/Ano1*^*fl/fl*^ mice, significantly (p < 0.05) shorter than those for control mice (Figure 2A).

Formalin is also a well-known algogenic chemical that produces nocifensive responses. Formalin-induced nocifensive behaviors are subdivided into two phases [30,31]. The first phase of nocifensive behaviors that lasts for 10 min after the formalin injection is caused by direct stimulation of sensory nerves whereas the second phase starting 10 min after formalin injection is mediated by inflammatory reaction [30,31]. As shown in Figure 2B, ablation of *Ano1* in DRG neurons is resulted in a significant decrease in the duration of nocifensive behaviors in the 2^nd^ phase of nociception after formalin injection (230 ± 46 *vs* 123 ± 15.9 s, p < 0.05, n = 6–7). In contrast, *Adv/Ano1*^*fl/fl*^ mice failed to show the difference in the duration of nocifensive behaviors in the first phase (0–10 min) after formalin injection when compared to that of control mice. Thus, these results further indicate that ANO1 is involved in mediating BK- or formalin-induced inflammatory pain.

### Reduction in neuropathic hyperalgesia and allodynia in Adv/Ano1^fl/fl^ mice

**Figure 3:**
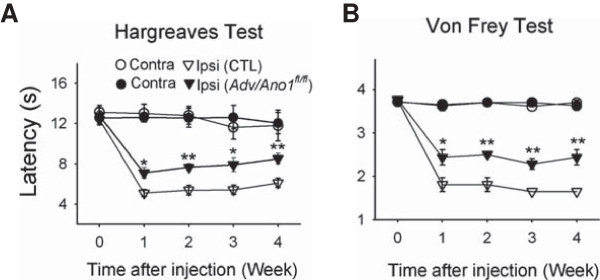
**Ablation of ANO1 in DRG neurons reduces spinal nerve injury-induced thermal and mechanical hyperalgesia. (A)** Neuropathic pain was induced on a hind paw after dissecting two out of three branches in the sciatic nerve. Paw withdrawal latencies of ipsilateral (Ipsi) and contralateral (contra) hind paws of *Adv/Ano1*^*fl/fl*^ and control mice were measured in response to radiant heat after spared nerve injury (*p < 0.05, ***p < 0.001 compared to the withdrawal latency of ipsilateral hind paw of control mice, n = 5–6). Basal paw withdrawal latency was measured before the surgery. **(B)** Withdrawal thresholds of ipsilateral and contralateral hind paws of *Adv/Ano1*^*fl/fl*^ and control mice were measured with Von Frey hairs after spared nerve injury (*p < 0.05, **p < 0.01 compared to the withdrawal threshold of ipsilateral hind paw of control mice, n = 5–6).

We then examined whether ANO1 could contribute to neuropathic hyperalgesia and allodynia. A spared nerve injury (SNI) model was adopted to induce neuropathic pain after cutting 2 out of 3 branches of the sciatic nerve (see Methods). Hargreaves and Von Frey hair tests were performed in hind paws of control and *Adv/Ano1*^*fl/fl*^ mice on every week for a 4-week period after nerve injury. Starting from one-week after surgery, withdrawal latency from a radiant heat was dramatically reduced in ipsilateral paws of control mice (p < 0.001, one way-ANOVA, Duncan post-hoc test, n = 5) (Figure 3A). The withdrawal latency of ipsilateral paws of *Adv/Ano1*^*fl/fl*^ mice was also significantly reduced after the nerve injury (p < 0.001, one way-ANOVA, Duncan post-hoc test, n = 6) (Figure 3A). However, the reduction in the withdrawal latency was significantly smaller than that of control mice for all time points of measurement (p < 0.01 ~ 0.05, n = 5 – 6) (Figure 3A).

Through the significant reduction (p < 0.01, one way-ANOVA, Duncan post-hoc test, n = 6) in the withdrawal threshold to Von Frey hairs after the nerve injury, mechanical allodynia was evident in ipsilateral hind paws of control mice after the nerve injury (Figure 3B). The reduction in the withdrawal threshold was not observed in contralateral hind paws of control mice. Significant mechanical allodynia (p < 0.01, one way-ANOVA, Duncan post-hoc test, n = 6) was also evident in ipsilateral hind paws of *Adv/Ano1*^*fl/fl*^ mice. However, the reduction in the withdrawal threshold after nerve injury was significantly smaller than that of control mice (p < 0.01 ~ 0.05, n = 5–6, Figure 3B). These results indicate that ANO1 plays a role in thermal hyperalgesia and mechanical allodynia induced by nerve injury.

### ANO1 augments the excitability of DRG neurons

**Figure 4:**
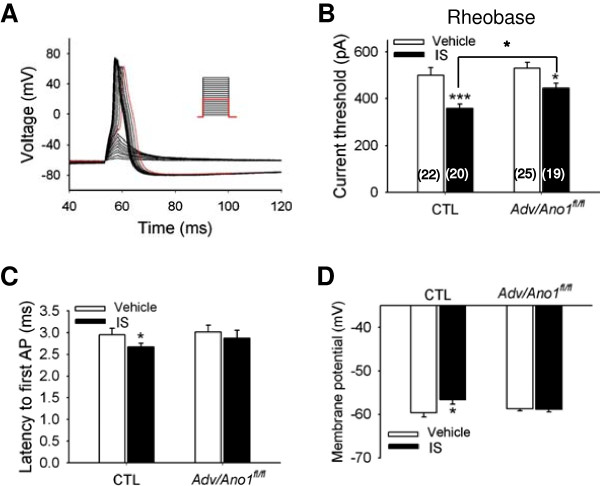
**Ablation of ANO1 in DRG neurons reduces inflammation-induced excitability of DRG neurons. (A)** Example traces of action potentials evoked by current injections to DRG neurons of control mice. Rheobase was determined as a current required for activating the first action potential (red). Depolarizing currents with 5 ms duration were injected to whole cells of DRG neurons from control mice from 50 pA to 1 nA with 50 pA increment (inset). Bath solution contained 140 mM NaCl whereas pipette solution contained 30 mM Cl^-^ and 50 nM free Ca^2+^ (see Methods). **(B)** Rheobases of DRG neurons isolated from *Adv/Ano1*^*fl/fl*^ and control mice were measured in the absence (vehicle) or presence of inflammatory soup (IS). Note that the rheobase was lowered in IS-treated DRG neurons both from control and *Adv/Ano1*^*fl/fl*^ mice. But, the reduction in the rheobase in IS- treated DRG neurons from *Adv/Ano1*^*fl/fl*^ mice was significantly less than that of control mice (*p < 0.05, one-way ANOVA, Duncan’s post-hoc test). ***p < 0.001, *p < 0.05 compared to the rheobase of the vehicle-treated DRG neurons. Numbers in parenthesis represent the number of neurons tested. **(C)** Latencies to first action potential (AP) were measured in DRG neurons of *Adv/Ano1*^*fl/fl*^ and control mice under the condition of absence or presence of inflammatory soup (IS). Current with 1 nA magnitude was injected to evoke the action potential spike. Note that the latency to the first AP was shortened in IS-treated DRG neurons from control mice whereas the latency to the first AP was not changed with IS treatment in DRG neurons from *Adv/Ano1*^*fl/fl*^ mice. *p < 0.05 compared to the latency of vehicle-treated DRG neurons. Number of neurons in each group is same as shown in B. **(D)** Resting membrane potentials of DRG neurons from *Adv/Ano1*^*fl/fl*^ and control mice were measured under the inflammatory condition. Number of neurons in each group is same as shown in B. *p < 0.05.

We then investigated whether ANO1 might contribute to the change in the excitability of DRG neurons under inflammatory condition. In order to determine changes in excitability, currents from 50 pA to 1 nA were injected to DRG neurons to measure the rheobase as a parameter for excitability, a minimal current that evokes an action potential [32,33]. In addition, latency to induce the first action potential was also measured when a current of 1 nA was injected to a DRG neuron as another parameter for excitability [34]. To imitate inflammatory condition, an inflammatory soup (IS), a mixture of four inflammatory mediators such as BK, prostaglandin E_2_, histamine, and serotonin was applied to primary-cultured DRG neurons [34,35]. The pipette solution contained 30 mM KCl^-^, 110 mM K-aspartate and 50 nM free Ca^2+^ whereas the bath solution contained 140 mM NaCl and 5 mM KCl. When the vehicle was applied to DRG neurons isolated from control mice, the rheobase was 500.0 ± 32.3 pA (n = 22) (Figure 4B). However, when DRG neurons were incubated with the IS for 2 hr, the rheobase was significantly decreased to 357.5 ± 18.3 pA (p < 0.001, n = 20), suggesting an increased excitability after the IS treatment. When DRG neurons from *Adv/Ano1*^*fl/fl*^ mice were incubated with IS, the rheobase was also significantly reduced (530.0 ± 23.9 (n = 25) vs 444.7 ± 20.7 pA (n = 19), p < 0.05). However, the reduction in the rheobase in the IS- treated DRG neurons from *Adv/Ano1*^*fl/fl*^ mice was significantly less than that of control mice (*p < 0.05, one-way ANOVA, Duncan’s post-hoc test) (Figure 4B). Similarly, the IS application to DRG neurons of control mice reduced a latency to induce the first action potential after 1 nA current injection significantly (p < 0.05) when compared to that of vehicle-treated DRG neurons (Figure 4C). In contrast, the IS application failed to change the latency for the induction of the first action potential in DRG neurons from *Adv/Ano1*^*fl/fl*^ mice (Figure 4C). The resting membrane potential of DRG neurons isolated from control mice depolarized significantly from -59.6 ± 0.9 mV to -56.7 ± 0.9 mV (p < 0.05) after the IS treatment, suggesting the increased excitability in the resting state (Figure 4D). In contrast, the resting membrane potential of DRG neurons isolated from *Adv/Ano1*^*fl/fl*^ mice was not affected by the IS treatment (Figure 4D). These results indicate that ANO1 augments excitability of DRG neurons during inflammation.

## Discussion

ANO1 is activated by intracellular Ca^2+^ and mediates many physiological functions as a candidate gene for Ca^2+^-activated Cl^-^ channel [16-18,20,21,36]. Mostly, ANO1 is present in nociceptors of DRG neurons [16,23]. Remarkably, ANO1 is activated by heat over 44°C and mediates acute thermal pain, because *Ano1*-deficient mice displayed reduced nociceptive behaviors in response to heat [23]. The present study extends its role from a heat transducer to a regulator of transmission of nociceptive signals evoked by inflammation or neuropathy. Indeed, ANO1 plays an active role in mediating inflammatory as well as neuropathic pain because inflammation or nerve-injury induced hyperalgesia or allodynia was reduced in *Adv/Ano1*^*fl/fl*^ mice. The decreased excitability in *Adv/Ano1*^*fl/fl*^ mice may account for the apparent reduced hyperalgesia and allodynia during inflammation. Thus, in addition to its ability of transducing acute noxious thermal stimuli to nociceptive neural signals, ANO1 is susceptible to intracellular signals that shape or affect transmission of nociceptive signals to the spinal cord during pathological conditions.

Thermal or mechanical hyperalgesia and allodynia are common features of neuropathic pain or inflammatory pain [1,5]. Pathological pain occurs in response to various inflammatory mediators such as prostaglandins, histamine, BK, ATP, serotonin (5-HT), proton, cytokines, growth factors, and neuropeptides that are released from inflammed or injured tissues [37]. These chemicals evoke peripheral sensitization, resulting in enhancement of the excitability of nociceptive nerve fibers. Several intracellular signaling pathways are known to cause the peripheral sensitization [38]. Some inflammatory agents such as BK, prostaglandins, ATP or endothelin-1 activate various protein kinases and modulate channels via phosphorylation [39-42]. The target channels would be TRPV1, TRPA1, and Nav1.8 that are expressed in terminals of nociceptors [43,44]. Similarly, ANO1 is highly expressed in nociceptors and activated by heat and also has multiple protein kinase consensus sites for protein kinase A, casein kinase, and other protein kinases [16,23]. Because ANO1 regulates the inflammation-induced membrane excitability in DRG neurons (Figure 4), phosphorylation of ANO1 by several kinases may account for the change of excitability of sensory neurons caused by inflammation.

Many thermoTRP channels are activated by multiple stimuli such as their respective ligands, voltage, and changes in temperature, which often produce synergistic effect among each other on their activities [7,45]. Responses of TRPV1 by heat and capsaicin were markedly potentiated by moderate acid that normally does not induce any TRPV1 current. In addition, TRPM3 has a strong synergistic effect between heat and pregnenolone sulfate, a neuroactive steroid [45]. The temperature-response curve of TRPM3 was shifted leftward by pregnenolone sulfate. Similarly, activity of ANO1 by heat is markedly augmented by its endogenous ligand, Ca^2+^. The increase in intracellular Ca^2+^ enhances heat-evoked ANO1 currents, decreasing the temperature threshold of ANO1 below 44°C [23]. For instance, when intracellular Ca^2+^ was increased above 0.5 μM, ANO1 was activated by the temperature close to body temperature, 37.5°C. Because inflamed tissues can cause the increase in intracellular Ca^2+^[2,46], it is conceivable that ANO1 can be sensitized by heat at body temperature under such a pathological condition.

It has been suggested that the regulation of intracellular Cl^-^ concentration in sensory neurons is involved in the mediation of inflammatory hyperalgesia or transmission of nociceptive signals [47,48]. Numerous studies have explained that the accumulation of intracellular Cl^-^ by NKCC1 which cause Cl^-^ uptake is correlated with pain behaviors. Nociceptive behaviors induced by formalin injection were significantly attenuated by NKCC1 blockers, bumetanide and furosemide [49]. In addition, itch and flare responses by histamine injection were also reduced by these NKCC1 blockers [50,51]. If intracellular Cl^-^ concentration is increased in inflamed tissues, the driving force of Cl^-^ to depolarization becomes greater when ANO1 is activated. Thus, an increase in intracellular Cl^-^ concentration during inflammation would also contribute to the ANO1′s role in mediating chronic pain.

In summary, the present study revealed that *Ano1* deletion in DRG neurons reduced inflammation and neuropathy-induced hyperalgesia and allodynia. In addition, the sensitization of DRG neurons induced by inflammation mediators was also absent in *Ano1*-ablated DRG neurons. These results suggest that ANO1 contributes to the sensitization of nociceptors during pathological conditions. The involvement of ANO1 in regulating the inflammatory or nerve-injury evoked pain may lead to the possibility that antagonists of ANO1 can be developed as novel analgesics.

## Competing interests

The authors declare that they have no competing interests.

## Authors’ contribution

BL constructed and bred knock out mice, carried out behavioral tests and electrophysiology. HC carried out behavioral tests and electrophysiology. JJ carried out behavioral tests. YDY genotyped knock out mice. DJY carried out behavioral tests. UO designed experiments and wrote the manuscript. All authors read and approved the final manuscript.
